# The Irradiation of Motor Impulses

**Published:** 1887-06

**Authors:** 


### SELECTIONS.

### The Irradiation of Motor Impulses.

If a man performs work with the muscles of, for example, his right hand exclusively, and to the point of fatigue, can he thereafter perform as much work of the same nature, with the left hand, as he could if the right hand had not been previously exercised? Such is the question discussed by Dr. N. A. Randolph before the College of Physicians of Philadelphia on March 2d. The question does not relate to a comparison of the work of the two hands, but to an examination of the work which may be done by one hand as conditioned by the previous exercise or non-exercise of its fellow; and it is clear that the answer to the question depends solely on intracranial processes, and that the answer would throw light on the functional independence or interdependence of the two halves of the brain. And to answer the question two conditions are prerequisite: the subject of the experiment must be ignorant of the object of the investigation, as otherwise he will be unconsciously a partisan of one hand, and there must also be a strong inducement for him to exercise his volition to the utmost.

Suitable subjects were found in the persons of some of the more vigorous and intelligent convicts of the Eastern Penitentiary, the stimulus being a money prize to the one who performed the most work in a given time. In the first series of experiments rubber-bulb syringes, identical in all their measurements, were used, the work performed being estimated by the amount of water which the men could transfer from one vessel to another in a given time. The uniform result of some forty observations was that either hand could do more work when its exercise preceded than when it followed the similar exercise of its fellow. "It was found, however, that the muscular effort could not be entirely restricted to one side of the body in this method, as great fatigue was always accompanied by a grimacing and writhing which implicated the muscles of both sides of the face and trunk." In the next experiments a Morse telegraph instrument was used, the muscular movements being restricted to an up-and-down motion of one finger of each hand, the number of such movements in a given time being recorded on the telegraph slip as dots or dashes, in accordance with the rapidity of contraction and relaxation of the flexors of the fingers. The results of these experiments were uniformly confirmatory of those in the first experiment; but the method caused such eye-strain in counting that it was abandoned. A clock-work instrument was then made, so that the records could be made on a dial and easily noted. Six subjects were used, and to each was given fifteen minutes in which to make his record with, for example the right fore-finger, after which the left fore-finger was similarly exercised. The same process was repeated on the following day, but beginning with the finger of the hand used second on the previous day.

These results were also practically uniform. "The man who for fifteen minutes flexed and relaxed his right forefinger with the greatest speed possible to him would, on the following day, accomplish on an average, nearly 10 per cent. less work with that finger when its exercise was consecutive to a similar exercise of the forefinger of the opposite side, than when its work was initial." As a rule, more work was accomplished by the simultaneous exercise of the two forefingers than by their successive exercise. In such simultaneous exercise of both hands, apparently from some unconscious effort at rhythm, it was seen that the movements of the left forefinger were generally more active and rapid than when used alone. This is in accordance with experience of many pianists. It will be seen that these facts are closely related to the observations of Weir Mitchell and Morris Lewis in regard to the knee-jerk. It will be remembered that they have shown that the knee-jerk is reinforced by any voluntary movement in any part of the body, and that this reinforcement is apparently due to such an irradiation of motor impulses from the active centres to other similar centres, as placed them and their related muscles in a condition of heightened responsiveness to external stimuli. Dr. Randolph's experiments seem to confirm this, and show that fatigue of any one centre may induce a sympathetic fatigue in other centres. These observations further suggest that the centres for volition, attention, and coordination are not, as regards their functional activity, bilaterally symmetrical and independent; that is to say, "these functions have not attained complete differentiation into right and left will, attention, or coordination; that, probably, the first effect of the voluntary activity of a portion of one cortical motor area is a stimulation of the corresponding portion of the other hemisphere—a stimulation that may result in its slightly premature fatigue; that apparently more work can be effected through the voluntary simultaneous exercise of two such portions of the motor apparatus than by their independent exercise one after the other."

Can we explain Dr. Randolph's facts by saying that the overflow of energy on to symmetrically related centres, or on to others, is competent to weaken them without being strong enough to cause motion? Dr. Weir Mitchell inclines toward the use of the overflow theory to explain the lowered capacity for work by one hand after exhaustion of the other. It will explain why in consentaneous use of two symmetrical parts more work is done than when they follow one the other. The overflow would be in this case valuable, and not damaging or wasteful. He referred to the fact, which he and Dr. Lewis have established, that "When we use the maximum power of one hand on a dynamometer, the co-instantaneous use of the other hand adds nothing to the result; and this form of experiment has been commonly used as a test of the reinforcing capacity of the opposite member. If, however, using two fingers, or the grip of the thigh adductors, on the bulb of a mercurial dynamometer until great exhaustion occurs, and we then make a new effort at the moment of violent use of another member, the mercury leaps quick to the level attained during the first effort by unfatigued muscles. It does not seem easy to explain this fact, except by assuming that the overflow of energy usually wasted is in this case made efficient."

This experiment brings up the question of muscular and ganglionic tone. When we strike the patellar tendon, a sudden, distant, voluntary act adds reinforcement. What happens to the muscle or ganglion so influenced? Is it made more sensitive to impressions, or is there with this a slight flow upon it of motor energy? And if so, can we measure the effect, and thus influence what we conceive of as muscular tone? Dr. Mitchell has been engaged in discovering if these reinforcements do cause motion—*i. e.*, a slight preparatory muscular contraction making the subsequent volition, or other excitatory activity, more potent in its results. He has been able, so far, to prove that in some spastic cases distant muscular effort, such as a grimace, really causes distinct and measurable movement in the extensors of the thigh and presumably elsewhere. But it is not yet clear whether in *normal* man remote motion is thus capable of causing slight shortening of all other muscles. We often speak, says Dr. Mitchell, of nerve power as if there were a common stock from which to draw the supplies needed by every active organ, and reason that it is unwise to try to carry on at once two functions which exact large expenditures—as digestion and intense thought, or digestion and exercise. Practically the difficulty may be one chiefly of blood supply. This is illustrated in the not rare fact that some feeble people cannot digest except when at rest. These facts suggest the idea that perhaps Dr. Randolph's cases would lose 10 per cent. of mechanical activity after a period of exhausting mental labor or during digestion.

In regard to the old theory for explaining the principle of counter-irritation, that there is a certain amount of nerve force in the system, and that when by means of counter-irritation the nerve force is drawn to a distant point, it is removed from the inflamed part, we may agree with Dr. H. C. Wood that, while modern science does not recognize the truth of this theory, it looks as though there is a certain amount of truth in it. " Every one who has worked in a gymnasium will recall the fact that he cannot use the two hands simultaneously with the same force as he can when the two hands are used separately. This shows some relation between the nerve centres which we have not as yet got at." It would seem that when we use our muscles vigorously, two kinds of fatigue are produced; a local fatigue and a general fatigue. If a man uses the right arm vigorously, he not only fatigues the right arm, but also the whole body. Dr. Wood believes that if these investigations are continued, it will be found that after prolonged use of the leg, there will be loss of power in the arm, perhaps as great as after previous use of the other arm. As suggested by Dr. Mitchell, "the relation between muscular exertion and mental exercise should be studied. Each one knows by personal experience that when mentally fatigued he is incapable of performing the usual amount of physical labor. This is probably independent of any question of overflow, and goes back to the higher cerebral centres and their relation." As suggested by Dr. Mills, it is a well-known clinical fact that an old hemiplegic, if examined carefully, will be found to have not only the decided loss of power and accompanying conditions resulting from the lesion on the opposite side of the brain, but also a certain diminution of strength in the limbs of the other side; a condition which is not entirely due to the general loss of physical power present. The phenomena exhibited in certain cases of unilateral spasm seem also to be related to this subject.

Experiments such as those of Dr. Randolph are not only of great interest in connection with pure physiology, but also in connection with psycho-physiology, or physiological psychology, which is now receiving more and more attention, and on which Prof. Ladd, of Yale, has recently written a most valuable book. —*Journal American Medical Association.*

